# Low-dose decitabine priming with intermediate-dose cytarabine followed by umbilical cord blood infusion as consolidation therapy for elderly patients with acute myeloid leukemia: a phase II single-arm study

**DOI:** 10.1186/s12885-019-5975-8

**Published:** 2019-08-20

**Authors:** Xiaoyang Li, Yuexin Dong, Ya Li, Ruibao Ren, Wen Wu, Hongming Zhu, Yunxiang Zhang, Jiong Hu, Junmin Li

**Affiliations:** 1 Shanghai Institute of Hematology, Ruijin Hospital Affiliated to Shanghai Jiao Tong University School of Medicine, 197 Ruijin No.2 Road, Shanghai, 200025 People’s Republic of China; 2 Department of Hematology, Ruijin Hospital Affiliated to Shanghai Jiao Tong University School of Medicine, Shanghai, China; 3 Shanghai Jiao Tong University School of Medicine, Shanghai, China

**Keywords:** Acute myeloid leukemia, Decitabine, Umbilical cord blood

## Abstract

**Background:**

Treatment of acute myeloid leukemia (AML) in elderly patients remains a great challenge. In this prospective single arm study (ChiCTR-OPC-15006492), we evaluated the efficacy and safety of a novel consolidation therapy with low-dose decitabine (LD-DAC) priming with intermediate-dose cytarabine (ID-Ara-C) followed by umbilical cord blood (UCB) infusion in elderly patients with AML.

**Methods:**

A total of 25 patients with a median age of 64-years-old (60–74-years-old) who achieved complete remission (CR) after induction chemotherapy were enrolled in the study.

**Results:**

The 2-year actual overall survival (OS) rate and leukemia-free survival (LFS) was 68.0 and 60.0%, respectively. The hematological and non-hematological toxicity were mild to moderate, and only one patient died in remission due to infection with possible acute graft versus host disease (aGVHD). Compared to a concurrent cohort of patients receiving conventional consolidation therapy, the study group tended to have an improved OS and LFS (*p* = 0.046 and 0.057, respectively), while the toxicity was comparable between the two groups.

**Conclusions:**

This study suggested the novel combination of LD-DAC, ID-Ara-C, and UCB infusion might be an optimal consolidation therapy for elderly patients with AML, and a prospective phase III randomized study is warranted to confirm this observation.

**Trial registration:**

This single-arm phase II clinical trial in elderly AML patients was registered prospectively at www.chictr.org.cn (identifier: ChiCTR-OPC-15006492) on June 2, 2015.

**Electronic supplementary material:**

The online version of this article (10.1186/s12885-019-5975-8) contains supplementary material, which is available to authorized users.

## Background

Acute myeloid leukemia (AML) is a common type of leukemia in adults, especially in older adults [1]. Although the overall survival (OS) of patients with AML has improved in the past three decades, this improvement has been limited to younger patients [2]. Several underlying factors contribute to poor outcomes, such as unfavorable cytogenetic abnormalities or mutation profiles, drug resistance, and intolerance of standard chemotherapy in elderly AML patients [3, 4]. Several attempts have been made to optimize the consolidation chemotherapy, but the overall outcome remains unsatisfactory [5–10]. Allogeneic hematopoietic stem cell transplantation (Allo-HSCT) has become the standard therapeutic option for AML patients after complete remission. However, not all patients are eligible for HSCT, and a sizable proportion of patients may eventually die of transplantation-related mortality. Also, in a clinical setting, AML patients ≥60 years old are rarely provided with the option of HSCT. As a result, it is crucial to develop novel post-remission treatment strategies in elderly patients with AML.

Outside the setting of classical allo-HSCT, infusion of HLA-mismatched granulocyte colony-stimulating factor (G-CSF) mobilized peripheral blood cells combined with intensive chemotherapy has demonstrated promising clinical outcomes in AML patients [11]. Nevertheless, verification of these results is warranted. T cells in umbilical cord blood (UCB), an alternative source for allo-HSCT, were reported to possess superior anti-tumor effects compared with adult peripheral T cells [12]. In 2012, Majhail et al. [13] reported that UCB is a feasible option for AML/myelodysplastic syndrome (MDS) patients in the absence of suitable donors. Later, the same group retrospectively investigated the outcome of 10 AML/MDS patients older than 70-years-old who received UCB transplantation and reported a 2-year OS of 60%, similar to those who received HLA full-matched sibling donor transplantation [14].

Hypomethylating agents (HMAs), such as decitabine (DAC) or azacytidine, have demonstrated encouraging results in the treatment of myelodysplasia syndrome and more recently AML in elderly patients, especially among those who are not eligible for intensive chemotherapy. In addition, the combination of HMAs and chemotherapy agents may have synergistic effects in the induction of leukemia cell apoptosis, while augmenting natural killer (NK) cell responsiveness [15] and tumor specific cytotoxic T lymphocyte responses [16], as demonstrated in animal models and in human cells. Clinical studies have also shown that administration of decitabine prior to chemotherapy increases responsiveness [17] and results in higher complete remission (CR) rates [18]. Therefore, the addition of HMAs to cytoreductive treatment before stem cell infusion might improve the overall outcome.

Based on the aforementioned studies, we developed a novel consolidation therapy consisting of low-dose decitabine (LD-DAC) and priming intermediate-dose cytarabine (ID-Ara-C) combined with UCB infusion in elderly patients with AML. Here, we report its efficacy and safety in a single-arm phase II study.

## Methods

### Study enrollment and oversight

This was an investigator-initiated, prospective, nonrandomized, single-arm phase II clinical trial in elderly AML patients registered at www.chictr.org.cn (identifier: ChiCTR-OPC-15006492). The study was approved by the Human Ethics Committee of the Ruijin Hospital and was in accordance with the Declaration of Helsinki. Written informed consent was obtained from all patients. Decitabine was provided free of charge by Chiatai Tianqing Pharma (China), which played no role in the study design, data collection, analysis, or writing of the manuscript.

Elderly patients with newly diagnosed AML, including MDS transformed AML or secondary AML, were eligible to participate in the study. Patients with acute promyelocytic leukemia or with a blast crisis of chronic myeloid leukemia were excluded. The induction chemotherapy consisted of daunorubicin hydrochloride (45 mg/m^2^) or idarubicin hydrochloride (8 mg/m^2^) for 3 days in combination with cytarabine (100 mg/m^2^) for 7 days, which was given for 1 or 2 cycles. After induction therapy, only those patients with documented CR defined according to standard criteria were enrolled in the study. Other inclusion criteria included an age of 60–75-years-old with an Eastern Cooperative Oncology Group (ECOG) performance status (PS) of 0–3.

### Interventions

#### Treatment protocol

The post-remission therapy included two cycles of low-dose decitabine [15 mg/m^2^ intravenously over 4 h for 5 consecutive days (day 1–5)], intermediate-dose cytarabine [1.0 g/m^2^ at q12 h for 2 days (day 6–7)] followed by infusion of one unit of UCB on day 9. Decitabine was provided free of charge by Chiatai Tianqing Pharma (China), which played no role in the study design, data collection, analysis, or writing of the manuscript. No immunosuppression was given as prophylaxis for graft versus host disease (GVHD), unless acute GVHD (aGVHD) was documented or clinically diagnosed. Infection prophylaxis and other support treatments, such as G-CSF, was administered according to a regular transplantation program. The comorbidities were assessed with the hematopoietic cell transplantation comorbidity index (HCT-CI) before each cycle of treatment. The second cycles of treatment were repeated in up to 2-month intervals. After two courses of post-remission therapy, patients received 1 course of Etoposide (100 mg intravenously for 5 consecutive days) and cytarabine (100 mg intravenously for 5 consecutive days), followed by 2 cycles of 6-mercaptopurine (25 mg/d d1–14), all-trans-retinoic acid (20 mg bid d29–56), and vitamin D3 (125 iu/d d57–84) sequential treatment as maintenance treatment.

The standard care for AML patients achieving first CR (CR1) at Ruijin Hospital is three courses of chemotherapy with Ara-C 0.5–1.0 g/m^2^ intravenously every 12 h for 3 days. We also collected clinical follow-up data of 24 elderly AML patients who received this traditional chemotherapy after CR1 (traditional chemotherapy group, TCG) for comparison of the clinical outcomes.

#### Selection of umbilical cord blood

High-resolution HLA typing for HLA-A, B, and DR loci were performed in all enrolled patients. The UCB units were obtained from the cord blood bank at the China Cord Blood Bank Network if they [1] were serologically matched for four or five of six HLAs, and [2] contained at least 3 × 10^7^ nucleated cells/kg of recipient body weight before freezing (Additional file 1: Table S1).

#### Chimerism analysis

After treatment, peripheral-blood cells were obtained from all participants and then tested for chimerism by standard cytogenetic analysis and a semi-quantitative PCR-based analysis of the short tandem repeats with a sensitivity of 1%.

#### Monitoring of minimal residual disease

The detailed MRD detection process was described previously [19]. Monoclonal antibodies against 20 antigens as follows: CD34, CD38, CD117, HLA-DR, CD13, CD33, CD14, CD15, CD64, CD11b, CD7, CD56, CD2, CD4, CD19, MPO, TdT, cyCD3, cyCD79a, and CD45, were utilized. Leukemia associated immunophenotyping (LAIPs) was classified at diagnosis with different surface antigens. A cut-off value of 0.1% was set as minimal residual disease (MRD).

### Study endpoints

The primary endpoint of our study is the 2-year leukemia-free survival (LFS). Secondary end points consisted of the 2-year OS, the incidence of hematological and non-hematological toxicity, median time to the recovery of neutropenia or platelets, incidence of aGVHD or chronic GVHD (cGVHD), 2-year incidence of treatment-related mortality (TRM), and documentation of chimerism in blood mononucleated cells. The LFS, OS, TRM, aGVHD and cGVHD were evaluated according to published criteria [20–22]. The time to hematopoietic recovery was determined as the duration from the end of chemotherapy to the time when the neutrophil count was > 0.5 × 10^9^/L and when the platelet count was > 20 × 10^9^/L without transfusion.

### Study design and statistical analysis

We used the Simon’s two-stage optimal design for this phase II study [23]. We expected this novel treatment protocol might decrease or delay relapse and result in an improved LFS. A 25% increase in LFS was of interest to further a large-scale phase III clinical trial. Based on the study design, 11 patients will be accrued in the first phase, and if 6 patients or less remained in continuous remission, the study will be stopped. Otherwise, 14 additional patients will be accrued for a total of 25 patients. The null hypothesis will be rejected if 17 or more patients remain in LFS at 1 year. This design yields a type I error rate of 0.05 and power of 0.8. Severe toxicity of the treatment is closely monitored. The accrual of patients will be halted if excessive numbers of patients die in remission during the treatment until the last follow-up, that is, if the number of patients died in remission is equal to or exceeds *bn* out of n patients with full follow-up according to the Pocock-type stopping boundary (as shown in Additional file 2: Table S2) [24]. Data in the study were statistically analyzed using the Statistical Package for Social Science (SPSS version 22.0). Survival curves were plotted using the Kaplan-Meier method. A *p* value of less than 0.05 was considered statistically significant.

## Results

### Patients’ characteristics

From January 2015 to May 2017, a total of 25 patients 60–74-years-old with AML in CR1 were enrolled in the study according to the patient’s willingness to participate in this study (Table 1). The diagnoses were defined according to the French-American-British and World Health Organization criteria [20]. Cytogenetic studies on pretreatment bone marrow samples were performed according to the International System of Human Cytogenetic Nomenclature [25]. Screening for molecular markers AML1-ETO, CBFβ-MYH11, NPM1, FLT3-ITD, FLT3-TKD, CEBPA, MLL-PTD, TET2, N-RAS, and DNMT3A was performed, and the prognostic risk groups were defined according to the NLE 2017 criteria [26].

**Table 1:** Clinical characteristics of patients

	UCB	TCG	*P* value
Patients, *n*	25	24	
Median age, years (range)	64.0 (60–73)	64.1 (60–71)	
Gender, *n*			0.656
Male	12	10	
Female	13	14	
FAB subtypes, *n*			0.779
M0-M1	3	2	
M2	11	12	
M4	5	4	
M5	4	5	
M6-M7	2	1	
AML, de novo	23	23	
Secondary to MDS	2	1	
ECOG			0.527
0–1	24	22	
≥ 2	1	2	
HCT-CI score			0.876
0	11	10	
1/2	12	10	
≥ 3	2	4	
ELN 2017 risk classification, *n*			0.463
Favorable	5	4	
Intermediate	14	15	
Adverse	6	4	
Molecular aberrant, n/Na	14/25	17/24	
FLT3-ITD	1	2	
FLT3-TKD	1	1	
NPM1	5	7	
DNMT3A	3	2	
CEBPA	3	6	
AML-ETO	2	1	
MLL	3	1	
N-RAS	1	2	
MRD to induction, *n*			0.879
≤ 0. 1%	13	13	
> 0. 1%	12	11	
Neutrophils≥0.5 × 10^9^/L, d, median (range)	11.9 (0–19)	15.3 (2–26)	0.028
Platelet ≥20 × 10^9^/L, d, median (range)	11.5 (8–18)	18.8 (11–28)	0.009

*Abbreviations*: *CBG* Cord blood group, *TCG* Traditional chemotherapy group, *FAB* French-American-British, *ECOG* Eastern Cooperative Oncology Group, *HCT-CI* Hematopoietic cell transplantation comorbidity index, *ELN* European Leukemia Net, *MRD* Minimal residual disease, *n/Na* The number of patients with gene mutations/the number of patients with molecular genetics examination, some patients have ≥2 gene mutations

Twenty-four patients in TCG were also listed in Table 1. Overall, there was no significant difference in the patients’ characteristics except for consolidation chemotherapy.

### Overall outcome

Upon the latest follow-up schedule as of May 2019, all patients have been followed-up for at least 2 years or met the primary endpoint. Fifteen of 25 patients remained in CR1, while 10 patients relapsed at a median of 16.5 months (range 4–32). Eight patients died of relapsed AML, and only one patient died of infection with possible aGVHD on day 20 after the first cycle of UCB treatment, which was characterized by persistent fever with antibiotics coverage, skin rash, liver function damage, and eventually development of multi-organ failure (MOF). The median OS and LFS for all patients was 31.9 months (range 4–53 months) and 29 months (range 4–53 months), respectively, with an actual 2-year OS and LFS at 68.0 and 60.0%, respectively.

**Fig. 1:**
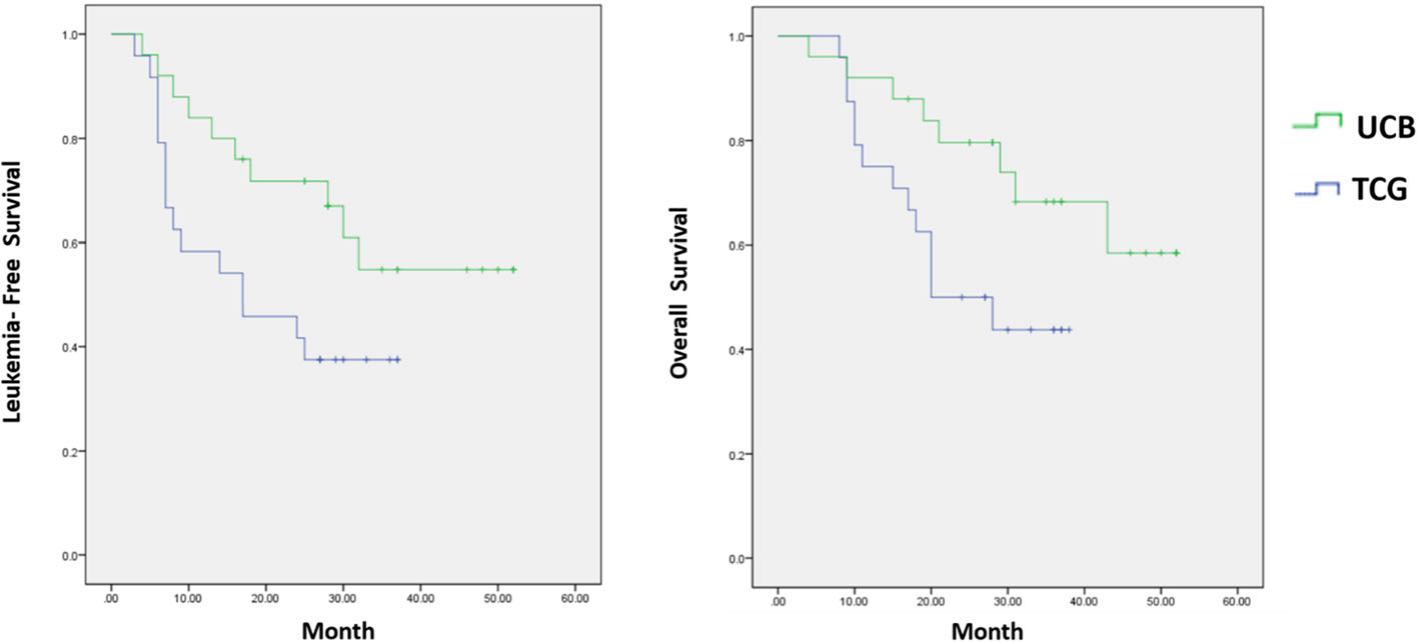
**a** Leukemia-free survival according to treatment. The 2-year LFS was 60 and 37.5%, respectively, in the UCB and TCG group (*p* = 0.057). **b** Overall survival according to treatment. The 2-year OS was 68 and 45.8%, respectively, in the UCB and TCG group (*p* = 0.046)

As to the overall outcome, the actual 2-year OS (45.8%) and LFS (37.5%) in the TCG was inferior to the study group (*p* = 0.046 and 0.057, respectively) as shown in Fig. 1.

### Hematological and non-hematological toxicities

The grade III to IV hematological toxicities including neutropenia and thrombocytopenia were common after treatment in both groups (as shown in Table 2). However, no hemorrhage was documented, and severe infection was diagnosed only in few cases (*n* = 2 in UCB and *n* = 3 in TCG). Also, the study group had an earlier hematological recovery than TCG. The median time to the recovery of the neutrophils was 11.9 days and 15.3 days, respectively, in UCB and TCG groups (*p* = 0.028). The recovery of platelets was also faster in the UCB group (11.5 days) compared to the TCG (18.8 days, *p* = 0.009).

**Table 2:** Toxicities^a^

	UCB n (%)		TCG n (%)	
	Grade 1–2	Grade 3–4	Grade 1–2	Grade 3–4
Hematological Toxicity				
Trombocytopenia	2 (8)	23 (92)	1 (4.1)	23 (95.8)
Neutropenia	10 (40)	15 (60)	16 (66.7)	8 (33.3)
Neutropenic fever	2 (8)	1 (4)	5 (20.8)	2 (8.3)
Anemia	19 (76)	6 (24)	20 (83.3)	3 (12.5)
Non Hematological Toxicity				
Hepatobiliary disorders	0	1 (4)	2 (8.3)	0
Mucositis	5 (20)	0	4 (16.7)	0
Skin disorders	2 (8)	0	1 (4.1)	0
Cardiac disorders	3 (12)	0	4 (16.7)	0
Sepsis	2 (8)	0	3 (12.5)	0
aGVHD	0	1 (4)	0	0

*Abbreviations*: *aGVHD* Acute graft versus host disease

^a^The severity of adverse events was graded on a scale of 1–5 according to the NCI Common Terminology Criteria for Adverse Events (NCI-CTCAE) v4.0

Non-hematological toxicities were documented in up to 20% of patients, but were usually mild to moderate. In the UCB group, 1 patient experienced liver function damage, 5 experienced mucositis disorder, 2 had skin rash, and 3 were diagnosed cardiac disorder. Severe infection (sepsis) was documented in only 2 patients in the UCB group, and 1 patient developed infection together with clinically diagnosed aGVHD and eventually died of MOF, as shown in Table 2. Non-hematological toxicities were comparable between the UCB group and TCG.

### Chimerism and GVHD

The chimerism was regularly tested on day 7 after UBC infusion. Of the 25 patients, only one patient (4%) had an established mixed chimerism level at 56.7%. For the remaining 24 patients, 20 (83.3%) had a very low level of micro-chimerism, with a range of 0.003–0.171%. For GVHD, only the aforementioned patient with a high level of mixed chimerism developed clinical signs of grade III aGVHD after UCB infusion and eventually died of infection and MOF 20 days after UCB infusion. No definite clinical aGVHD or cGVHD was observed in any other patients.

### Treatment outcome by MRD level

In this study, we monitored the treatment response in patients by detecting LAIPs in bone marrow through flow cytometry after induction therapy, each cycle of consolidation therapy, and every 3 months afterwards. Patients with low MRD (≤ 0.1%) after induction therapy tend to have a better LFS in the study group compared to patients with an MRD level over 0.1% (76.9% vs. 41.7%, *p* = 0.173). Intriguingly, for patients maintaining a high MRD level (≥ 0.1%) after CR1, the 2-year LFS was significantly higher in the UCB group (41.7%) compared to those treated with conventional consolidation therapy (9.1%, *p* = 0.008)

## Discussion

Although 50–60% of elderly AML patients can achieve CR with induction therapy, the consolidation was not well established. Conventional chemotherapy tends to have a poor outcome with a 2-year OS of about 10–20%. Juliusson et al. [27] reported the use of cytarabine in elderly AML patients as a consolidation therapy, and the 2-year OS and LFS remained at 25 and 22%, respectively. Recently, allo-HSCT in elderly AML patients became more prevalent in a clinical setting. Kasanon et al. [28] reported a 3-year OS of 38% in elderly AML patients receiving T-cell-repleted haploidentical HSCT.

Recent studies reported that cord blood T cells exert superior anti-tumor effects compared with adult peripheral blood T cells [12]. The novel micro-transplantation strategy with the infusion of G-CSF-mobilized HLA-mismatched donor peripheral-blood stem cells following high-dose cytarabine has also shown an encouraging outcome in the treatment AML, including elderly AML, with a 2-year OS and LFS of 50.2 and 42.1%, respectively [29].

Inspired by these reports of cellular therapy, we evaluated the efficacy and safety of a novel consolidation regimen consisting of low-dose decitabine priming with an intermediate dose cytarabine followed by UCB infusion. The inclusion of decitabine in the protocol was based on the evidence of previous studies. First, it has been shown that the sequence and combination of decitabine and cytarabine present synergy in the induction of cell apoptosis in leukemia cell lines [30]. Second, in myeloid leukemia cell lines, transient, low-dose decitabine exposure has been shown to induce CD80 gene expression in a variety of human leukemia cells, which provides evidence that epigenetic modulation can induce the expression of a major T cell co-stimulatory molecule on cancer cells, may overcome immune tolerance, and induce an efficient anti-tumor response [16]. More recently, in mice challenged with myeloid leukemia celli line THP-1, decitabine was the only hypomethylation agent that may enhance the anti-AML effect of CD34+ derived NK cells. In a clinical setting, two retrospective studies demonstrated that decitabine combined with cytarabine or low-dose chemotherapy, followed by the infusion of G-CSF mobilized peripheral blood, potentially improved the treatment outcomes in elderly AML patients [31, 32]. Interestingly, recent study reported that T cells from UCB possess more anti-leukemia potential than T cells from adult peripheral T cells. On the other hand, UCB may exert effects in promoting hematopoiesis, modulating NK cell responsiveness [33, 34], and was associated with a reduced risk of GVHD compared to mobilized peripheral blood, while maintaining similar graft-versus-leukemia effects (GVL) in HSCT settings [34]. Another important reason to choose UCB rather than G-CSF mobilized peripheral blood from haplo-identical family donors is to save the potential donors for future salvage of allo-HSCT.

In the present study, we reported a 2-year OS of 68.0% and a 2-year LFS of 60.0% in older patients with AML, while only 1 patient died in remission. The hematological and non-hematological toxicities were mostly mild to moderate, which were also comparable to conventional consolidation chemotherapy. Of greater interest, we noticed that patients receiving the novel protocol tend to have a more rapid recovery of platelet counts and significantly higher platelet levels after recovery. The explanation might be the protective effects of LD-DAC on megakaryocytes and its maturation and platelet production, as shown in the mouse model, or possible unidentified effects conferred by UCB [35]. This may provide an important advantage in the novel regimen, because the improvement of both OS and LFS may be attributed to the fact that the novel consolidation was more tolerable to elderly patients, while the standard consolidation chemotherapy may cause slow hematopoietic recovery, leading to a delay in the treatment schedule and/or dose reduction, thus impeding the overall outcome. Therefore, we considered that the novel treatment is feasible and perhaps the more favorable choice for the treatment of elderly patients with AML.

As MRD monitoring plays an important role in AML [36–39], we also regularly monitored the bone marrow (BM) MRD level by flow cytometry. Patients with low bone marrow MRD levels (≤ 0.1%) immediately after induction therapy tend to have a better LFS. However, this difference is not statistically significant, probably due to the limited number of patients. Of note, when we compared the outcome of patients who failed to obtain the low MRD level after induction therapy, the 2-year LFS was significantly higher in the study group (41.7%) compared to those treated with conventional consolidation therapy (9.1%, *p* = 0.008). Therefore, our data suggest that patients with higher MRD levels after induction therapy can still benefit from LD-DAC combined chemotherapy with UCB infusion. During subsequent MRD monitoring after 2 cycles, LD-DAC chemotherapy combined with UCB treatment showed a reduced relapse potential in patients who achieved low MRD levels. This observation suggests LD-DAC chemotherapy combined with UCB infusion may achieve promising outcomes in patients who achieved low MRD levels early after induction or consolidation therapy. However, longer follow-up is required to confirm this observation.

## Conclusion

Based on the study design, our data suggests that LD-DAC combined with ID-Ara-C with UCB infusion has the potential to improve the overall outcome in elderly AML patients. One limitation of the study is the small sample size and relative short follow-up. A prospective, randomized, phase 3 confirmatory study with a sufficient number of patients with long follow-up is warranted to further confirm our observation; a multi-center study is underway to compare the long-term outcomes of the novel approach versus conventional chemotherapy consolidation in elderly AML patients.

## Additional files

Additional file 1: **Table S1.** HLA match status from patient to donor. (DOCX 17 kb)

Additional file 2: **Table S2.** Continuous monitoring for severe toxicity by Pocock-type boundary. (DOCX 14 kb)

## Data Availability

The datasets used and/or analyzed during the current study are available from the corresponding author upon reasonable request.

## Abbreviations

aGVHD: Acute graft versus host disease
Allo-HSCT: Allogeneic hematopoietic stem cell transplantation
AML: Acute myeloid leukemia
BM: Bone marrow
cGVHD: Chronic GVHD
CR: Complete remission
DAC: Decitabine
ECOG: Eastern Cooperative Oncology Group
G-CSF: Ranulocyte colony-stimulating factor
GVHD: Graft versus host disease
GVL: Graft-versus-leukemia effect
HCT-CI: Hematopoietic cell transplantation comorbidity index
HMAs: Hypomethylating agents
ID-Ara-C: Intermediate-dose cytarabine
LAIPs: Leukemia associated immunophenotyping
LD-DAC: Low-dose decitabine
LFS: Leukemia-free survival
MDS: Myelodysplastic syndrome
MOF: Multi-organ failure
MRD: Minimal residual disease
NK: Natural killer
OS: Overall survival
PS: Performance status
TRM: Treatment-related mortality
UCB: Umbilical cord blood
