# Supplementary material for: Low-dose decitabine priming with intermediate-dose cytarabine followed by umbilical cord blood infusion as consolidation therapy for elderly patients with acute myeloid leukemia: a phase II single-arm study
Source: BMC Cancer. 2019 Aug 20;19:819. doi: 10.1186/s12885-019-5975-8 (PMC6701020; doi:10.1186/s12885-019-5975-8)
Supplement: Supplementary file 1 — Table S1. HLA match status from patient to donor. (DOCX 17 kb) [file 12885_2019_5975_MOESM1_ESM.docx]

| **Table S1 HLA match status from patient to donor** | | | | | |
| --- | --- | --- | --- | --- | --- |
| No. |  | HLA-A | HLA-B | HLA-DRB1 | match |
| 1 | Patient | 02;11 | 62;60 | 04;09 |  |
|  | Donor1 | 02;11 | 40;40 | 04;09 | 4/6 |
|  | Donor2 | 02;11 | 40;40 | 04;09 | 4/6 |
| 2 | Patient | 02;32 | 38;52 | 01;16 |  |
|  | Donor1 | 02;03 | 38;52 | 15;16 | 4/6 |
|  | Donor2 | 26;32 | 38;52 | 04;15 | 4/6 |
| 3 | Patient | 02;11 | 27;46 | 09;12 |  |
|  | Donor1 | 02;11 | 13;46 | 09;12 | 5/6 |
|  | Donor2 | 02;11 | 27;46 | 01;12 | 5/6 |
| 4 | Patient | 29;32 | 07;07 | 07;11 |  |
|  | Donor1 | 29;32 | 07;44 | 07;08 | 4/6 |
|  | Donor2 | 29;32 | 07;44 | 07;16 | 4/6 |
| 5 | Patient | 02;33 | 51;58 | 03;09 |  |
|  | Donor1 | 02;33 | 46;58 | 03;09 | 5/6 |
|  | Donor2 | 02;33 | 51;58 | 03;12 | 5/6 |
| 6 | Patient | 02;02 | 51;67 | 09;12 |  |
|  | Donor1 | 02;02 | 51;67 | 12;15 | 5/6 |
|  | Donor2 | 02;02 | 40;67 | 09;12 | 5/6 |
| 7 | Patient | 02;11; | 40;46 | 07;08 |  |
|  | Donor1 | 02;11 | 46;51 | 08;12 | 4/6 |
|  | Donor2 | 02;11 | 39;46 | 08;11 | 4/6 |
| 8 | Patient | 02;24 | 40;46 | 04;09 |  |
|  | Donor1 | 02;24 | 40;46 | 04;16 | 5/6 |
|  | Donor2 | 02;24 | 46;46 | 04;09 | 5/6 |
| 9 | Patient | 02;11 | 40;51 | 09;12 |  |
|  | Donor1 | 02;11 | 15;40 | 09;12 | 4/6 |
|  | Donor2 | 02;11 | 15;40 | 09;12 | 4/6 |
| 10 | Patient | 30;31 | 13;35 | 12;15 |  |
|  | Donor1 | 30;31 | 13;35 | 09;15 | 5/6 |
|  | Donor2 | 30;31 | 13;35 | 07;15 | 5/6 |
| 11 | Patient | 02;33 | 39;44 | 11;13 |  |
|  | Donor1 | 02;33 | 40;44 | 11;13 | 5/6 |
|  | Donor2 | 02;33 | 39;44 | 04;13 | 5/6 |
| 12 | z | 30;33 | 13;44 | 07;07 |  |
|  | Donor1 | 30;33 | 13;44 | 07;09 | 5/6 |
|  | Donor2 | 30;33 | 15/44 | 07;07 | 5/6 |
| 13 | Patient | 02;30 | 13;46 | 07;10 |  |
|  | Donor1 | 02;30 | 13;46 | 07;15 | 5/6 |
|  | Donor2 | 02;30 | 13;46 | 07;13 | 5/6 |
| 14 | Patient | 24;24 | 40;48 | 11;12 |  |
|  | Donor1 | 24;24 | 60;48 | 11;12 | 5/6 |
|  | Donor2 | 24.24 | 40;48 | 09;12 | 5/6 |
| 15 | Patient | 02;02 | 46;54 | 14;15 |  |
|  | Donor1 | 02;02 | 46;55 | 15;15 | 4/6 |
|  | Donor2 | 02;02 | 46;55 | 04;15 | 4/6 |
| 16 | Patient | 02;02 | 13;55 | 12;12 |  |
|  | Donor1 | 02;02 | 13;55 | 12;15 | 5/6 |
|  | Donor2 | 02;02 | 13;55 | 04;12 | 5/6 |
| 17 | Patient | 11;26 | 15;39 | 04;09 |  |
|  | Donor1 | 11;26 | 15;15 | 09;12 | 4/6 |
|  | Donor2 | 11;26 | 39;54 | 04;15 | 4/6 |
| 18 | Patient | 11;24 | 40;40 | 04;12 |  |
|  | Donor1 | 11;24 | 15;40 | 04;12 | 5/6 |
|  | Donor2 | 11;24 | 40;40 | 09;12 | 5/6 |
| 19 | Patient | 02;02 | 46;46 | 08;08 |  |
|  | Donor1 | 02;02 | 40;46 | 08;08 | 5/6 |
|  | Donor2 | 02;02 | 35;46 | 08;08 | 5/6 |
| 20 | Patient | 02.24 | 40;40 | 09;09 |  |
|  | Donor1 | 24;24 | 40;40 | 09;09 | 5/6 |
|  |  |  |  |  |  |
| 21 | Patient | 24;24 | 35;40 | 04;11 |  |
|  | Donor1 | 24;24 | 15;35 | 11;12 | 5/6 |
|  | Donor2 | 24;24 | 35;40 | 04;15 | 5/6 |
| 22 | Patient | 11;24 | 13;27 | 12;12 |  |
|  | Donor1 | 11;24 | 13;27 | 11;12 | 5/6 |
|  | Donor2 | 11;24 | 13;27 | 04;12 | 5/6 |
| 23 | Patient | 02;11 | 15;46 | 08;09 |  |
|  | Donor1 | 02;11 | 35;46 | 08;09 | 5/6 |
|  | Donor2 | 02;11 | 14;46 | 08;09 | 5/6 |
| 24 | Patient | 02;32 | 38;52 | 01;16 |  |
|  | Donor1 | 02;11 | 38;52 | 15;16 | 4/6 |
|  | Donor2 | 02;32 | 38;52 | 02;16 | 5/6 |
| 25 | Patient | 02;24 | 46;55 | 09;12 |  |
|  | Donor1 | 02;24 | 13;46 | 09;12 | 5/6 |
|  | Donor2 | 02;24 | 15;46 | 09;12 | 5/6 |
