# Supplementary material for: Low-dose decitabine priming with intermediate-dose cytarabine followed by umbilical cord blood infusion as consolidation therapy for elderly patients with acute myeloid leukemia: a phase II single-arm study
Source: BMC Cancer. 2019 Aug 20;19:819. doi: 10.1186/s12885-019-5975-8 (PMC6701020; doi:10.1186/s12885-019-5975-8)
Supplement: Supplementary file 2 — Table S2. Continuous monitoring for severe toxicity by Pocock-type boundary. (DOCX 14 kb) [file 12885_2019_5975_MOESM2_ESM.docx]

**Table S2: Continuous monitoring for severe toxicity by Pocock-type boundary***

| Number of Patients, *n* | 1 | 2 | 3 | 4 | 5 | 6 | 7 | 8 | 9 | 10 | 11 | 12 | 13 | 14 | 15 | 16 | 17 | 18 | 19 | 20 |
| --- | --- | --- | --- | --- | --- | --- | --- | --- | --- | --- | --- | --- | --- | --- | --- | --- | --- | --- | --- | --- |
| Boundary, *b_n_* | 1 | 2 | 2 | 2 | 2 | 2 | 2 | 2 | 2 | 3 | 3 | 3 | 3 | 3 | 3 | 3 | 3 | 3 | 3 | 3 |
| Number of Patients, *n* | 21 | 22 | 23 | 24 | 25 |  | | | | | | | | | | | | | | |
| Boundary, *b_n_* | 4 | 4 | 4 | 4 | 4 |  |  |  |  |  |  |  |  |  |  |  |  |  |  |  |

 * The trial will be stopped if the number of patients died in remission is equal to or exceeds bn out of n patients with completed follow-up.
